# Improved mycobacterial protein production using a *Mycobacterium smegmatis groEL1ΔC *expression strain

**DOI:** 10.1186/1472-6750-11-27

**Published:** 2011-03-25

**Authors:** Elke E Noens, Chris Williams, Madhankumar Anandhakrishnan, Christian Poulsen, Matthias T Ehebauer, Matthias Wilmanns

**Affiliations:** 1 European Molecular Biology Laboratory (EMBL), Hamburg Outstation, c/o DESY, Building 25a, Notkestraße 85, 22603 Hamburg, Germany; 2 The Scripps Research Institute, La Jolla, 92037, USA

## Abstract

**Background:**

The non-pathogenic bacterium *Mycobacterium smegmatis *is widely used as a near-native expression host for the purification of *Mycobacterium tuberculosis *proteins. Unfortunately, the Hsp60 chaperone GroEL1, which is relatively highly expressed, is often co-purified with polyhistidine-tagged recombinant proteins as a major contaminant when using this expression system. This is likely due to a histidine-rich C-terminus in GroEL1.

**Results:**

In order to improve purification efficiency and yield of polyhistidine-tagged mycobacterial target proteins, we created a mutant version of GroEL1 by removing the coding sequence for the histidine-rich C-terminus, termed GroEL1ΔC. GroEL1ΔC, which is a functional protein, is no longer able to bind nickel affinity beads. Using a selection of challenging test proteins, we show that GroEL1ΔC is no longer present in protein samples purified from the *groEL1ΔC *expression strain and demonstrate the feasibility and advantages of purifying and characterising proteins produced using this strain.

**Conclusions:**

This novel *Mycobacterium smegmatis *expression strain allows efficient expression and purification of mycobacterial proteins while concomitantly removing the troublesome contaminant GroEL1 and consequently increasing the speed and efficiency of protein purification.

## Background

Heterologous expression of recombinant proteins in *Escherichia coli *can result in the production of insoluble inclusion bodies. Recent statistics show that less than half of the *M. tuberculosis *(Mtb) proteins expressed in *E. coli *are soluble [1]. Therefore, the non-pathogenic bacterium *Mycobacterium smegmatis *is often used as an alternative, more closely related host for the expression of mycobacterial proteins. Furthermore, *M. smegmatis *may also provide mycobacterium-specific chaperones, which can help correct folding of Mtb proteins [1].

During nickel affinity purification, it has been observed that a protein of 56 kDa is co-purified with polyhistidine-tagged recombinant proteins while using *M. smegmatis *as an expression system. This contaminant was previously identified as the Hsp60 chaperone GroEL1 of *M. smegmatis *[1-3]. The protein sequence of GroEL1 shows a histidine-rich C-terminus (7 out of 11 amino acids are histidines), which is likely to be the reason for the observed nickel sepharose binding [1,2].

Unlike most other bacteria, mycobacteria possess two Hsp60 chaperone *groEL *genes, one of which is arranged in the bicistronic *groESL *operon [4]. *M. smegmatis *also encodes a third Hsp60 protein (Msmeg1978), which is more distantly related to GroEL1 (Msmeg1583) and GroEL2 (Msmeg0880) [3]. Although *groEL1 *of *M. smegmatis *can be found in the same operon as *groES*, an arrangement indispensable for the chaperone function in bacteria, its histidine-rich tail is distinct from the more typical glycine-methionine-rich C-terminal region found in GroEL2 [3]. Furthermore, *groEL2 *is an essential gene and exists in all actinobacteria, in contrast to *groEL1 *[3,5]. Recently, it has been shown that *groEL2 *and *groES *are expressed more strongly than *groEL1*, which might have arisen from a difference in stability of the predicted post-transcriptionally cleaved mRNAs for *groES *and *groEL1 *[5]. Consistent with the current chaperone model in mycobacteria, one chaperone, here GroEL2, would act as the main house keeping chaperone in *M. smegmatis*, with the other chaperones (GroEL1 and Msmeg1978) adopting more specialised functions. Indeed, GroEL1 of *M. tuberculosis *was recently identified as being associated with nucleotides, suggesting a role as a DNA chaperone, while GroEL1 of *M. smegmatis *was found to have a role in mycolic acid biosynthesis during biofilm formation [5,6,3].

The co-purification of GroEL1 with histidine-tagged recombinant proteins can be particularly problematic since native GroEL1 is expressed at relatively high levels, meaning that in the case of a low yield of recombinant protein, GroEL1 may well compete with the protein of interest for binding sites on nickel affinity beads. Minimal sample manipulation is recommended during protein purification to improve efficiency. Therefore, additional steps required to remove GroEL1 can result in a significant loss of the protein of interest.

In this article, we describe an *M. smegmatis *expression strain containing a mutant version of GroEL1, termed GroEL1ΔC, which consists of a *groEL1 *gene without a coding sequence for the histidine-rich C-terminal tail. We show that GroEL1ΔC is a functional protein, which no longer co-purifies when using nickel affinity purification and we provide evidence that proteins purified from this strain are correctly folded, active and that they behave identically to those purified from the original expression strain. Taken together, our data demonstrate that *M. smegmatis groEL1ΔC *is a competent protein expression strain, which allows the efficient removal of the troublesome contaminant GroEL1 without the requirement of additional purification steps.

## Methods

### Bacterial strains and media

The *E. coli *strains DH5α (Invitrogen) and HB101 (Promega) were used for cloning of expression constructs and the target substrate to generate the mutant version of *groEL1 *using standard procedures [7]. Transformants were selected in Luria Broth containing the appropriate antibiotics.

*M. smegmatis *mc^2^155 was used as the parent (wild type) strain for the *groEL1ΔC *strain. Both *M. smegmatis *strains were maintained in Middlebrook 7H9 or 7H10 medium supplemented with 0.2% (v/v) glycerol, 10% ADC, 0.05% (v/v) tween-80 and the appropriate antibiotics.

For biofilm formation, 10 ml of biofilm media was inoculated with 10 μl of saturated culture and incubated at 30°C without disturbance [3,8].

For the expression of the recombination proteins in *M. smegmatis *in order to create the mutant form of *groEL1*, 0.2% succinate (w/v) was added as a carbon source to 7H9 medium supplemented with 0.2% (v/v) glycerol, 0.05% (v/v) tween and the appropriate antibiotics. Expression of his-tagged recombinant proteins in *M. smegmatis *was performed in 7H9 medium supplemented with 0.2% (w/v) glucose as carbon source. Acetamide was added to a final concentration of 0.2% (w/v) at 0.5 OD_600 _and at 2.5 OD_600 _for the expression of the recombination proteins and his-tagged recombinant proteins, respectively.

### Plasmids, constructs and oligonucleotides

All plasmids and constructs are summarised in Table 1 and oligonucleotides are listed in Table 2. pJV53 was used to express the recombination proteins [9]. pYUB854 was used for the preparation of the target substrate to create the *groEL1ΔC *strain [10]. pGH542, harbouring a δγ resolvase, was used to generate an unmarked deletion [11]. Using the primer pairs Msmeg1583-F1.2 & Msmeg1583-R1 and Msmeg1583-F2 & Msmeg1583-R2.1, two 500 bp fragments, homologous to the fragments +1067/+1587 and +1621/+2176 relative to the translational start of Msmeg1583, were amplified and subsequently ligated *Afl*II-*Xba*I (F1.2-R1) and *Hind*III-*Spe*I (F2-R2.1) into pYUB854, creating pEN15.

**Table 1:** Plasmids and constructs used in this study

Plasmid/construct	Description	Reference
pJV53	Che9c recombination proteins under control of the acetamidase promoter in pLAM12	[9]

pYUB854	Hyg^R ^cassette flanked by γδ-res sites and 2 MCSs	[10]

pGH542	Expressing an γδ resolvase and tetracycline resistant	[11]

pEN15	pYUB854 with a 520 bp fragment harbouring g*roEL1 *(+1067/+1587, relative to *groEL1*) inserted upstream of the Hyg^R ^cassette and a 555 bp fragment downstream of *groEL1 *including the STOP codon of *groEL1*, inserted downstream of the Hyg^R ^cassette	This paper

pMyNT	Mycobacterial overexpression vector	Geerlof *et al.*, unpublished data

pMyNT/PrcA-B	Rv2109-2110 in pMYNT, Rv2110 is N-terminally his-tagged	[12]

pMyNT/AccD5E5	Rv3280-3281 in pMYNT. Only his-tagged Rv3280 seems to express using this construct.	This paper

pMyNT/AccA3	Rv3285 in pMyNT	This paper

pMyNT/CFP10-ESAT6	Rv3874-3875 in pMYNT, Rv3874 is N-terminally his-tagged	[12]

pMyNT/ACPS	Rv2523 in pMYNT	This paper

**Table 2:** Primers used in this study

Primer	Sequence (5'-3')	Location 5'	Relative to
Msmeg1583-F1.2	GCGC**CTTAAG**CGACTGGGATCGCGAGAAGCTGC	+1067	Msmeg1583

Msmeg1583-R1	GCGC**TCTAGA**CTCGTCCTCGTCGGCCGGCTTG	+1587	Msmeg1583

Msmeg1583-F2	GCGC**AAGCTT**GATCCATTTCACGCGACACCCCC	+1620	Msmeg1583

Msmeg1583-R2.1	GCGC**ACTAGT**GGTGTTCGATCGTCTGGCCGATG	+2176	Msmeg1583

accD5E5-F	GATC**TCATGA**GTATGACAAGCGTTACC G	+1	Rv3280

accD5E5-R	GTCA**AAGCTT**TTATCGGCGCATGTGCG	+2161	Rv3280

accA3-F	GATC**CCATGG**GTATGGCTAGTCACGCC	+2	Rv3285

accA3-R	GTCA**AAGCTT**TTACTTGATCTCGGCGAGC	+1803	Rv3285

Rv2523-F	CATG**CCATGG**GCATCGTCGGTGTGGGG	+1	Rv2523

Rv2523-R	CCC**AAGCTT**ACGGGGCCTCCAGGATGGC	+391	Rv2523

Restriction sites are presented in bold face. CTTAAG = *Eco*RI, TCTAGA = *Xba*I, CCATGG = NcoI, TCATGA = *Bsp*HI

AAGCTT = *Hin*dIII, ACTAGT = *Spe*I.

For the expression of *M. tuberculosis *proteins in *M. smegmatis*, the pMyNT expression vector was used [Geerlof *et al.*, unpublished data]. pMyNT/ACPS, pMyNT/AccA3 and pMyNT/AccD5 were made as follows: PCR was performed with primer pair Rv2523-F & Rv2523-R for ACPS, accA3-F & accA3-R for AccA3 and accD5E5-F & accD5E5-R for AccD5 and the resulting fragments were digested with *Nco*I-*Hin*dIII and inserted into *Nco*I-*Hin*dIII digested pMyNT.

### Creation of the *groEL1ΔC *mutant

The *groEL1ΔC *mutant was created using the mycobacterial recombineering method [9]. pEN15 was digested with *Afl*II and *Spe*I to create the linear target substrate, which was introduced into mc^2^155 electrocompetent cells, expressing the recombinase genes on pJV53 and in this way creating hygromycin-resistant transformants. The hygromycin-resistance cassette was removed using δγ resolvase, expressed on pGH542, generating an unmarked deletion [11].

### Southern blot analysis

Genomic DNA (5ug) was isolated as described [9], digested with the appropriate enzymes, separated on a 0.9% agarose gel and transferred to a positively charged nylon membrane (Roche). For DNA probe labelling, hybridisation and detection, the DIG high prime DNA labelling and detection starter kit 1 (Roche) was used.

### Growth curves

Bacterial growth was followed by measuring the optical densities at a wavelength of 600 nm as a function of time. Cultures were prepared with 7H9 expression medium (0.2% (w/v) glucose as carbon source) in identical triplicates for each strain. Duplicate samples were taken every 4 hours for 40 hours. When the optical density at 600 nm exceeded 1.5, samples were diluted in order to remain within the linear range of the detector.

### Protein expression and purification

All methods related to protein expression in *M. smegmatis *were carried out as described [12,13]. Protein-protein complexes from operon-encoded proteins were expressed using the native operon structure [9]. In brief, pellets from 500 ml cultures were dissolved in 30 ml lysis buffer containing 50 mM Tris-HCl pH 8.0, 300 mM NaCl, 0.5 M urea with protease inhibitor cocktail (Sigma) and 1 mg/ml DNase I (Serva). Resuspended cells were sonicated four times, each for 5 min (with a 0.3 s pulse and 0.7 s rest) at 5 min intervals to prevent overheating, using a Bandelin VW3200 probe at 45% amplitude. The supernatant was collected after centrifugation (30,000 × g) for 1 h at 4°C, filtered through a 0.44 μm filter and loaded onto a nickel affinity sepharose (NiAC) column. After washing with 10 column volumes of 50 mM Tris-HCl pH 8.0, 300 mM NaCl and 20 mM imidazole, proteins were eluted in 50 mM Tris-HCl, 100-150 mM NaCl and 250-500 mM imidazole and subjected to size exclusion chromatography using either a Superdex 75 (16/60) column (GE Healthcare) or, for large protein complexes, a Superose 6 (10/300) (GE Healthcare) with 25 mM Tris-HCl pH 8.0, 150 mM NaCl and 1 mM DTT as buffer. The collected protein samples were analysed by SDS-PAGE and concentrated accordingly.

### Circular Dichroism (CD) spectrum analysis

CD measurements were performed on a Jasco J-810 spectropolarimeter. Prior to measurement, samples were dialysed into 10 mM potassium phosphate, 150 mM NaCl, pH 7.4. Spectra were recorded between 182 and 260 nm in a 2 mm cuvette with machine settings as follows: 1 nm bandwidth, 1 sec response, 1 nm data pitch, 100 nm/min scan speed, cell length of 0.1 cm. Each curve presented is the average of three separate measurements.

### Coupled enzyme assay

Enzymatic activity of the AccD5-AccA3 complex was estimated by a coupled enzyme assay that follows the rate of ATP hydrolysis spectrophotometrically [14]. The production of ADP during the reaction was coupled to pyruvate kinase and lactate dehydrogenase, and the oxidation of NADH was probed at 340 nm. The assay mixture contained 7 units of pyruvate kinase, 10 units of lactate dehydrogenase, 50 mM NaHCO_3_, 3 mM ATP, 0.5 mM phosphoenol pyruvate, 0.2 mM NADH, 0.3 mg/ml BSA, 100 mM K_2_HPO_4 _pH 7.6 and 5 mM MgCl_2 _and varying concentrations of propionyl-coenzyme A. Reactions were initiated by the addition of enzyme to the assay mixture and were maintained at 30°C. Data were acquired using a Tecan infinite M1000 microplate reader. The kinetic parameters K_m _and V_max _were determined by fitting the mean velocities *versus *the substrate concentration to the Michaelis-Menten equation of enzyme kinetics using nonlinear regression analysis, executed by the program Prism 5 (GraphPad Software™).

## Results and Discussion

### Creation of the *groEL1ΔC *strain

**Figure 1:**
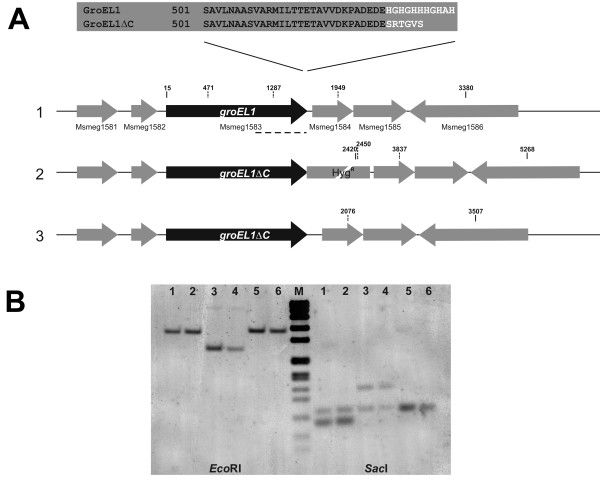
**Construction of the *groEL1ΔC *strain**. (A) A schematic representation of the genomic organisation of *groEL1 *(Msmeg1583) in *M. smegmatis *mc^2^155 (1), the hygromycin-resistant *groEL1ΔC *mutant (2) and the unmarked *groEL1ΔC *deletion strain (3). Msmeg1582 is *groES *and Msmeg1581, 1584, 1585 and 1586 encode for proteins of unknown function. Zoom sequence of the C-terminal 40 amino acids of the *groEL1 *gene product, showing the histidine-rich C-terminal region. In the *groEL1ΔC *strain, the eleven C-terminal amino acids (white) were replaced by six different residues. Hyg^R ^= hygromycin-resistance cassette. (B) Southern blot analysis performed with genomic DNA of *M. smegmatis *mc^2^155 (1), mc^2^155 carrying pJV53 (2), two correct hygromycin-resistant *groEL1ΔC *mutants (3-4) and two correct unmarked *groEL1ΔC *deletions (5-6). The genomic DNA was digested with *Eco*RI or *Sac*I. The positions of the restriction sites *Eco*RI and *Sac*I, relative to the start of *groEL1*, are presented as a full and dotted vertical line in A, respectively. The PCR product of primer pair Msmeg1583-F1.2 & Msmeg1583-R1 was used as a probe, shown as a dashed line under the genes in A1. (M) DNA marker VII, DIG labelled (Roche).

Currently, the role of GroEL1 in protein folding is uncertain. A closer look at the structure of *E. coli *GroEL [15] indicates that, although the C-terminal region of the protein is not easily accessible, pointing towards the central cavity of the wheel-like structure adopted by oligomeric GroEL, the extreme C-terminal 20 amino acids are absent from the model. Similarly, the GroEL structure of *Paracoccus denitrificans *also lacks these residues [16]. These observations suggest that the C-terminal region of GroEL is highly flexible and could reach out of the central cavity, allowing in this way *M. smegmatis *GroEL1 to bind nickel affinity beads. Additionally, as native GroEL1 from *M. tuberculosis *is oligomeric [17], nickel binding would require only one accessible histidine-rich region. Therefore, we decided to change only the last eleven amino acids of the protein, rather than to make a full knock out strain, in order to minimise changes to the expression strain. A precise chromosomal deletion of fragment 1588-1620, relative to the translational start of *groEL1 *(Msmeg1583), was created using the mycobacterial recombineering technique [9] (Figure 1A). Southern hybridisation (Figure 1B) was used to verify that correct homologous recombination had taken place in the hygromycin-resistant and the unmarked deletion strain (Figure 1B). The latter strain, in which the hygromycin resistance cassette has been removed, has the C-terminal eleven residues of GroEL1, containing seven histidines, replaced by six non-histidine residues, which are part of the "scar" sequence left behind after removal of the resistance cassette (Figure 1A). The stop codon of this recombinant version of GroEL1 is TAA, which although rare, is recognised in high G+C *mycobacteria *[18]. This unmarked deletion strain, referred to as *M. smegmatis groEL1ΔC*, is used in all further experiments.

**Figure 2:**
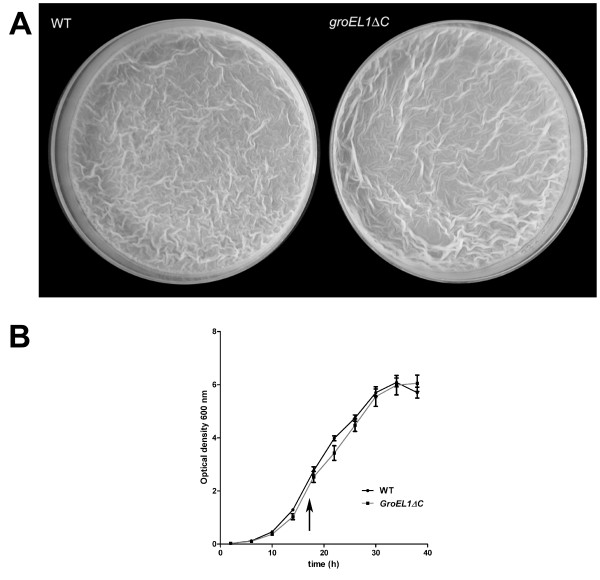
**Biofilm formation and growth rates of *M. smegmatis *mc^2 ^155 and *M. smegmatis groEL1ΔC *are comparable**. (A) Both *M. smegmatis *mc^2 ^155 (WT) and *M. smegmatis groEL1ΔC *strains are able to form biofilms after an incubation time of 7 days at 30°C. (B) Growth curve of *M. smegmatis *mc^2^155 (WT = black) and *M. smegmatis groEL1ΔC *strains (grey) in 7H9 expression medium. The arrow represents the typical time of induction in *M. smegmatis*.

Ojha *et al. *reported that the last 18 amino acids of GroEL1 are essential for the formation of mature biofilms [3]. Therefore, to test the functionality of the GroEL1ΔC protein, we compared biofilm formation in both the wild type and g*roEL1ΔC *strains. Both strains were able to form mature biofilms after an incubation time of 7 days at 30°C, indicating that GroEL1ΔC is indeed fully functional (Figure 2A). Taking into account the data from Ojha *et al.*, our results could suggest that either the amino acids important for biofilm formation are upstream of those removed in the GroEL1ΔC protein, or that removal of the last 18 residues may affect the folding of at least a part of GroEL1. Additionally, as this newly created strain was constructed for the overexpression of mycobacterial proteins, its growth in 7H9 expression medium was compared to the original expression strain *M. smegmatis *mc^2^155 (Figure 2B). We observed no significant differences in growth between the two strains, with both reaching an OD_600 _of between 2.5-3.0 after approximately 18 hours, at which time expression is usually induced.

### GroEL1ΔC is absent during nickel affinity purification of proteins expressed in *M. smegmatis groEL1ΔC*

**Figure 3:**
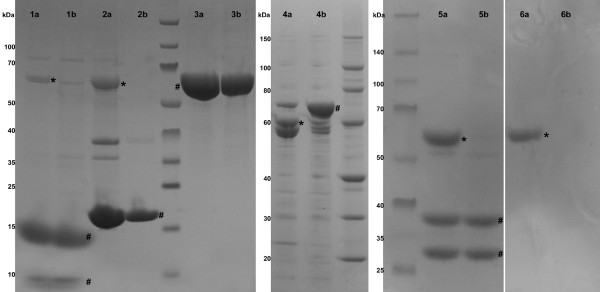
**GroEL1ΔC is absent from protein samples purified from *M. smegmatis groEL1ΔC***. SDS-PAGE analysis of protein samples isolated from cells expressing either CFP10-ESAT6 heterodimer (1), a holo-acyl-carrier protein synthase (ACPS) (2), the β- and α-subunit D5 (3) and A3 (4) of the acyl-CoA carboxylase AccD5-AccA3 complex, the PrcA-B complex (5) or the empty expression vector (empty pMyNT) (6). **a**, proteins expressed in *M. smegmatis *mc^2^155; **b**, proteins expressed in *M. smegmatis groEL1ΔC*. Purified proteins of interest are labelled with #. Visible presence of GroEL1 is depicted with *. Squares identify the bands excised for peptide mass fingerprinting. GroEL1 = 56 kDa.

To demonstrate the absence of GroEL1ΔC as a contaminant when using the *M. smegmatis groEL1ΔC *expression strain, we determined the expression and purification efficiency of our strain in comparison to the wild type strain using five different constructs, representing a variety of different protein molecules, including the mycobacterial proteasome, the CFP10-ESAT6 complex, the AccD5-AccA3 dodecameric acyl-CoA carboxylase complex and the holo-acyl-carrier protein synthase (for details, see Table 3). Additionally, we also used the empty pMyNT vector, to check for GroEL1 binding in the absence of a his-tagged protein. All constructs were transformed into both *M. smegmatis *mc^2^155 and *groEL1ΔC *and the resulting transformants were cultured in 7H9 expression medium and induced by the addition of acetamide to a final concentration of 35 mM. Eighteen hours after induction, the cells were collected by centrifugation, lysed and the soluble protein fraction was passed over a nickel affinity column, with the elution fraction being analysed by SDS-PAGE (Figure 3). While GroEL1 was visible in samples purified from *M. smegmatis *mc^2^155 (Figure 3, lanes a), the protein was noticeably absent in five out of six protein samples isolated from the *groEL1ΔC *strain (Figure 3, lanes b). Due to the fact that AccD5 has a similar size to GroEL1, we were unable to determine its presence or absence in samples of the purified acyl-CoA carboxylase complex by SDS-PAGE. Therefore, samples isolated from gel (Figure 3) were analyzed by mass spectrometry (Additional file 1). While numerous peptides from both GroEL1 and AccD5 could be identified from gel slices deriving from the mc^2^155 strain, only AccD5 peptides could be detected in the sample obtained from the *groEL1ΔC *strain (Additional file 1). Likewise, MALDI-TOF mass spectrometry was performed on the other protein samples, verifying the absence of GroEL1 peptides in the protein samples derived from *M. smegmatis groEL1ΔC *(data not shown).

**Table 3:** List of test proteins used to validate the *groEL1ΔC *expression strain

ORF		Annotation		Description	Expressed ...		Mol. Mass (kDa)
Rv2109c Rv2110c		PrcA PrcB		α- and β-subunit of the mycobacterial proteasome (α_7_β_7_β_7_α_7 _subunit organisation)	Using native operon content, producing a 730 kDa multimeric complex		26.8 30.3

Rv3285 Rv3280		AccA3 AccD5		α- and β-subunit from acyl-CoA carboxylase AccD5-AccA3 complex (α_3_β_3_β_3_α_3 _subunit organisation)	As monomeric proteins, mixed to form a acyl-CoA carboxylase complex of 740 kDa		63.8 59.4

Rv3874 Rv3875		CFP10 ESAT6		Potential virulence factor CFP10-ESAT6 complex	Using native operon content, producing a heterodimeric (1:1) complex		10.8 9.9

Rv2523c		ACPS		Holo-acyl-carrier protein synthase	As monomeric protein		14

### Proteins purified from *M. smegmatis groEL1ΔC *behave identically to those purified from the wild type strain

*M. smegmatis *encodes three forms of the Hsp60 chaperone GroEL: Msmeg1583 (GroEL1), Msmeg0880 (GroEL2) and Msmeg1978. However, the precise molecular function of each protein remains unclear. Changing the last 18 amino acids of GroEL1 does not alter growth but does result in a strong defect in biofilm formation [3]. To confirm that the newly created recombinant version of GroEL1 has no effect on the correct folding and, ultimately, the function of the proteins expressed in *M. smegmatis groEL1ΔC*, a number of different proteins and protein complexes have been expressed and analysed.

In the previous section, we have shown that it is possible to express and purify potentially challenging protein complexes, such as the proteasome complex PrcA-B and the CFP10-ESAT6 complex, from the recombinant *groEL1ΔC *strain. These data imply that the proteins isolated from the *groEL1ΔC *strain are correctly folded, since we were able to observe all components after purification. In both examples, complex formation requires direct protein-protein interactions between subunits of the complex as only one subunit is his-tagged.

Taking our analysis one step further, we directly tested the structural and functional properties of proteins isolated from the *groEL1ΔC *strain. We used the five expression constructs described above and transformed them into both *M. smegmatis *mc^2^155 and *groEL1ΔC*. Proteins were expressed and purified using a nickel affinity column as described above. AccD5 and AccA3 protein samples were mixed in a 1:1 stoichiometry to form the high-molecular-weight AccD5-AccA3 complex. Size exclusion chromatography was performed on all samples as a final purification step.

**Figure 4:**
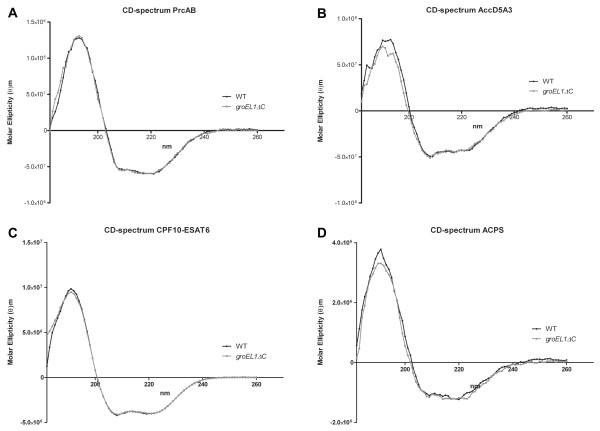
**Proteins isolated from both strains give virtually identical CD spectra**. CD spectra of the multimeric proteasome complex PrcA-B (A), the dodecameric acyl-CoA carboxylase AccD5-AccA3 complex (B), CFP10-ESAT6 heterodimer (C), and monomeric protein ACPS (D) expressed in *M. smegmatis *mc^2^155 (WT = black) and *M. smegmatis groEL1ΔC *(grey) are virtually identical. For A and B, a concentration between 170 and 200 nM was used while for C and D, concentrations were between 5 and 10 μM.

Circular dichroism (CD) spectroscopy is a powerful tool used to visualise the secondary structure properties of protein samples. We observed that the four protein samples isolated from g*roEL1ΔC *gave virtually identical CD spectra to those purified from the wild type strain (Figure 4), implying that they are correctly folded. Furthermore, the CD spectra of the CFP10-ESAT6 complexes, showing a protein with high helical content, are comparable to those collected previously [12] and are in line with the X-ray structure, which consists of a four-helical bundle complex (PDB ID: 3FAV) [12].

**Figure 5:**
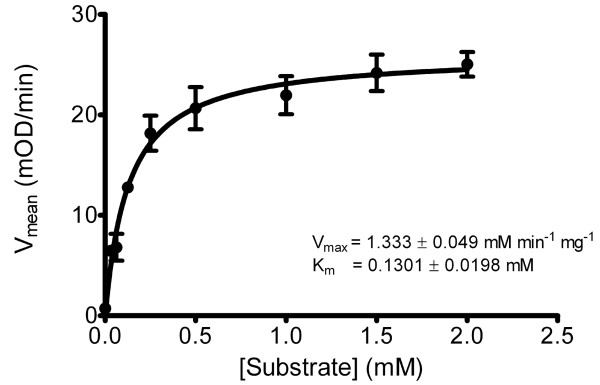
**Kinetics of AccD5-AccA3 isolated from *M. smegmatis groEL1ΔC***. Carboxylation activity of the acyl-CoA carboxylase AccD5-AccA3 complex isolated from *groEL1ΔC *was measured using an enzyme-coupled reaction with propionyl-CoA as substrate, providing Km = 0.1469 mM and Vmax = 28.5 mOD/min.

Additionally, we have demonstrated carboxylase activity of the acyl-CoA carboxylase AccD5-AccA3 complex, isolated from *groEL1ΔC*, using an enzyme-coupled reaction (Figure 5). Using propionyl-CoA as a substrate, AccD5-AccA3 showed carboxylase activity with a Km = 0.1301 ± 0.0198 mM and a Vmax = 1.333 ± 0.049 mM min^-1 ^mg^-1^, data which are similar to the parameters determined using the AccD5-AccA3 complex isolated from *E. coli *[19], indicating that the AccD5-AccA3 complex isolated from *groEL1ΔC *is a functional carboxylase. Carboxylase activity requires the α-subunit of the carboxylase to be post-translationally biotinylated [19], implying that the subunits of this large megasynthase are folded correctly and, in the case of the α-subunit, correctly post-translationally modified, when isolated from *groEL1ΔC*.

## Conclusions

We have developed an *M. smegmatis *expression strain that allows efficient expression and purification of mycobacterial proteins, multi-subunit protein complexes and post-translationally modified proteins while concomitantly removing the troublesome contaminant GroEL1 and consequently increasing the speed and efficiency of protein purification. The *M. smegmatis groEL1ΔC *strain is particularly suitable for laboratories performing *in vitro *activity assays and structural studies on mycobacterial proteins and protein complexes.

## Abbreviations

PCR: Polymerase chain reaction; kDa: kilo Dalton; Hsp60: Heat shock protein 60; ADC: Albumine-dextrose-catalase; DMSO: dymethylsulfoxide; NiAc: Nickel affinity sepharose column; SDS-PAGE: sodium dodecyl sulfate polyacrylamide gel electroporesis; MALDI-TOF: matrix-assisted laser desorption/ioization reflection time-of-flight.

## Authors' contributions

EN and CP designed the study. EN made the *groEL1ΔC *strain, tested its functionality and growth, expressed and purified all proteins described except the AccD5-AccA3 complex and wrote the manuscript. CW carried out the CD measurements, provided technical assistance and participated in writing the manuscript. MA carried out all experiments concerning AccD5-AccA3. CP provided expression constructs. ME participated in testing the strain's growth and feasibility. MW organized the funding, supervised the work and helped revising the manuscript. All authors read and approved the final manuscript.

## Supplementary Material

Additional file 1 **GroEL1 is absent from an AccD5 protein sample derived from *M. smegmatis groEL1ΔC***. Results of peptide mass fingerprinting analysis of samples excised from SDS-PAGE gel (Figure 3, boxes). Shown in red are the peptides that could be identified. (a) Sample derived from *M. smegmatis *mc^2^155. (b) Sample derived from *M. smegmatis groEL1ΔC*. Click here for file
