# Supplementary material for: Improved mycobacterial protein production using a Mycobacterium smegmatis groEL1ΔC expression strain
Source: BMC Biotechnol. 2011 Mar 25;11:27. doi: 10.1186/1472-6750-11-27 (PMC3076238; doi:10.1186/1472-6750-11-27)
Supplement: Additional file 1 — GroEL1 is absent from an AccD5 protein sample derived from M. smegmatis groEL1ΔC. Results of peptide mass fingerprinting analysis of samples excised from SDS-PAGE gel (Figure 3, boxes). Shown in red are the peptides that could be identified. (a) Sample derived from M. smegmatis mc2155. (b) Sample derived from M. smegmatis groEL1ΔC. [file 1472-6750-11-27-S1.PDF]

Figure S1

AccD5 (Rv3280) 90% coverage

MTSVTDRSAHSAERSTEHTIDIHTTAGKLAELHKRREESLHPVGEDAVEKVHAKGKLTARERIYALLDEDSFVELDALAKHRSTNFNLGEKRPLG
DGVVTGYGTIDGRDVCIFSQDATVFGGSLGEVYGEKIVKVQELAIKTGRPLIGINDGAGARIQEGVVSLGLYSRIFRNNILASGVIPQISLIMGAAA
GGHVYSPALTDFVIMVDQTSQMFITGPDVIKTVTGEEVTMEELGGAHTHMAKSGTAHYAASGEQDAFDYVRELLSYLPPNNSTDAPRYQAAAP
TGPIEENLTDEDLELDTLIPDSPNQPYDMHEVITRLLDDEFLEIQAGYAQNIVVGFGRIDGRPVGIVANQPTHFAGCLDINASEKAARFVRTCDCF
NIPIVMLVDVPGFLPGTDQEYNGIIRRGAKLLYAYGEATVPKITVITRKAYGGAYCVMGSKDMGCDVNLAWPTAQIAVMGASGAVGFVYRQQLA
EAAANGEDIDKLRLRLQQEYEDTLVNPYVAAERGYVDAVIPPSHTRGYIGTALRLLERKIAQLPPKKHGNVPL

GroEL (Msmeg1583) 30% coverage

M S K Q I E F N E T A R R A M E A G V D K L A D A V K V T L G P R G R H V V L A K S F G G P Q V T N D G V T I A R E I D L E D P Y E
N L G A Q L V K S V A T K T N D V A G D G T T T A T V L A Q A L V R A G L R N V A A G A N P I A L G S G I S K A A D A V S E A L L A
S A T P V D D K K A I A Q V A T V S S R D E Q V G E L V G E A M T K V G H D G V V T V E E S S T L E T Y L E V T E G V G F D K G F L
S A Y F V T D F D S Q E A V L E D A L V L L H R D K I S S L P D L L P L L E K V A E A G K P L L I V A E D V E G E A L S T L V V N A I R
K T L K A V A V K A P F F G D R R K A F L D D L A I V T G G Q V V N P D V G L L L R E V G L E V L G S A R R V V V N K D S T V I V D
G G G T A E A I A D R V K Q I K S E I E T T D S D W D R E K L Q E R L A K L A G G V A V I K V G A A T E T D L K K R K E A V E D A V A
A A K A A V E E G I V T G G G A A L V Q A R S A V E K L R G E L S G D E A L G V D V F A S A L S A P L Y W I A T N A G L D G S V V V
N K V S E L P K G Q G F N A A T L E F G D L V S A G V V D P A K V T R S A V L N A A S V A R M I L T T E T A V V D K P A D E D E H G
H G H H H G H A H

AccD5 (Rv3280) 63% coverage

MTSVTDRSAHSAERSTEHTIDIHTTAGKLAELHKRREESLHPVGEDAVEKVHAKGKLTARERIYALLDEDSFVELDALAKHRSTNFNLGEKRPLG
DGVVTGYGTIDGRDVCIFSQDATVFGGSLGEVYGEKIVKVQELAIKTGRPLIGINDGAGARIQEGVVSLGLYSRIFRNNILASGVIPQISLIMGAAA
GGHVYSPALTDFVIMVDQTSQMFITGPDVIKTVTGEEVTMEELGGAHTHMAKSGTAHYAASGEQDAFDYVRELLSYLPPNNSTDAPRYQAAAP
TGPIEENLTDEDLELDTLIPDSPNQPYDMHEVITRLLDDEFLEIQAGYAQNIVVGFGRIDGRPVGIVANQPTHFAGCLDINASEKAARFVRTCDCF
NIPIVMLVDVPGFLPGTDQEYNGIIRRGAKLLYAYGEATVPKITVITRKAYGGAYCVMGSKDMGCDVNLAWPTAQIAVMGASGAVGFVYRQQLA
EAAANGEDIDKLRLRLQQEYEDTLVNPYVAAERGYVDAVIPPSHTRGYIGTALRLLERKIAQLPPKKHGNVPL

GroEL (Msmeg1583) 0% coverage

M S K Q I E F N E T A R R A M E A G V D K L A D A V K V T L G P R G R H V V L A K S F G G P Q V T N D G V T I A R E I D L E D P Y E
N L G A Q L V K S V A T K T N D V A G D G T T T A T V L A Q A L V R A G L R N V A A G A N P I A L G S G I S K A A D A V S E A L L A
S A T P V D D K K A I A Q V A T V S S R D E Q V G E L V G E A M T K V G H D G V V T V E E S S T L E T Y L E V T E G V G F D K G F L
S A Y F V T D F D S Q E A V L E D A L V L L H R D K I S S L P D L L P L L E K V A E A G K P L L I V A E D V E G E A L S T L V V N A I R
K T L K A V A V K A P F F G D R R K A F L D D L A I V T G G Q V V N P D V G L L L R E V G L E V L G S A R R V V V N K D S T V I V D
G G G T A E A I A D R V K Q I K S E I E T T D S D W D R E K L Q E R L A K L A G G V A V I K V G A A T E T D L K K R K E A V E D A V A
A A K A A V E E G I V T G G G A A L V Q A R S A V E K L R G E L S G D E A L G V D V F A S A L S A P L Y W I A T N A G L D G S V V V
N K V S E L P K G Q G F N A A T L E F G D L V S A G V V D P A K V T R S A V L N A A S V A R M I L T T E T A V V D K P A D E D E H G
H G H H H G H A H
